# Mothers’ prenatal tobacco smoke exposure is positively associated with the occurrence of developmental coordination disorder among children aged 3–6 years: A cross-sectional study in a rural area of Shanghai, China

**DOI:** 10.18332/tid/119115

**Published:** 2020-03-27

**Authors:** Qing Yang, Liqin Pan, Cuili Shen, Huilin Yao, Qingqing Zhu, Chunfen Cheng, Ruiping Wang

**Affiliations:** 1 Songjiang Maternal and Child Health-care Hospital, Shanghai, China; 2 Songjiang Huating Kindergarten, Shanghai, China; 3 Office of Clinical Research Center, Yueyang Hospital of Integrated Traditional Chinese and Western Medicine, Shanghai University of Traditional Chinese Medicine, Shanghai, China

**Keywords:** prenatal tobacco smoke exposure, firsthand smoke, secondhand smoke, developmental coordination disorder, association

## Abstract

**INTRODUCTION:**

Prenatal tobacco smoke exposure is a potential risk factor for developmental coordination disorder (DCD) in children, but evidence on the relationship between DCD in children and tobacco smoke exposure in women is limited in China. We conducted a cross-sectional study to understand the prevalence of prenatal tobacco smoke exposure among mothers and the prevalence of DCD among children and to explore if mothers’ prenatal tobacco smoke exposure is positively associated with the occurrence of DCD among children.

**METHODS:**

During 2018, we sampled 8586 children aged 3–6 years and their mothers in Songjiang district, Shanghai. DCD in children was identified by MABC-2 screening combined with pediatrician confirmation. Prenatal tobacco smoke exposure among mothers was classified into firsthand smoke (FHS) and secondhand smoke (SHS) exposure. SAS 9.2 software was applied to calculate the prevalence of DCD in children, the prevalence of prenatal FHS and SHS exposure in mothers and to analyze the differences by chi-squared test and logistic regression.

**RESULTS:**

Among the 8586 women, the prevalence of prenatal FHS and SHS exposure was 0.94% and 20.79%, respectively. The prevalence of DCD in children was 6.65%, which was significantly higher in boys (7.64%) than in girls (5.51%), and children aged 3 years (9.77%) had a higher prevalence of DCD than children aged 4, 5 or 6 years (7.44%, 5.27% and 4.28%, respectively). In comparison with children whose mother was not exposed to prenatal FHS or SHS, the odds of having DCD was higher in children whose mother was exposed to prenatal FHS (OR=4.42; 95% CI: 2.62–7.44) and SHS (OR=1.77; 95% CI: 1.47–2.14), even after adjustment for potential confounders.

**CONCLUSIONS:**

The occurrence of DCD among children is positively associated with prenatal tobacco smoke exposure among the mothers. It is crucial to implement tobacco control measures to decrease the prevalence of smoking among pregnant women and SHS exposure at home as well as in the work environment.

**ABBREVIATIONS:**

DCD: developmental coordination disorder, ADHD: attention deficit hyperactivity disorder, FHS: firsthand smoke, SHS: secondhand smoke, MABC-2: movement assessment battery for children-2nd edition, GATS: global adult tobacco survey, SD: standard deviation, OR: odds ratio, CI: confidence interval, DAGs: directed acyclic graphs.

## INTRODUCTION

Developmental coordination disorder (DCD) is defined as a neurodevelopmental disorder and characterized by a marked impairment and clumsiness in the development of motor coordination abilities^1,2^. DCD not only interferes with activities of daily living but also affects children’s academic achievement along with emotional and behavioral issues^3,4^. Globally, the prevalence of DCD ranges from 1.8% to 8.6% among primary school children^5^, and ranges from 5.5% to 8.3% among children of preschool age^6,7^. In China, a study with 3316 children in Suzhou demonstrated that the prevalence of DCD was 7.6%^6^. Previous research have shown that the risk of DCD increases among children from less advantaged socioeconomic backgrounds, among children with a birthweight under 2500 g or born before 37 weeks of gestation^2,8,9^. Furthermore, boys have higher risk of developing DCD than girls, and the prevalence of DCD decreases when children become older^10,11^. In recent years, the increased prevalence as well as the risk factors of DCD among children have made it an important public health issue worldwide.

Maternal smoking during pregnancy is the most prevalent preventable cause of low birthweight, neonatal morbidity and premature delivery^12^. It has been well established that maternal smoking is a significant risk factor for attention deficit hyperactivity disorder (ADHD), which has an almost 50% overlap with DCD^13^, but the association of maternal smoking during pregnancy and DCD among children is equivocal^14,15^. A study with 122 children from Canada indicated that children exposed to secondhand smoke *in utero* have 3 times increased risk of DCD^16^, while another study with 744 children in Auckland, New Zealand, demonstrated that children whose mothers smoke during pregnancy have an increased risk of DCD in comparison with children whose mothers are non-smokers^17^. However, two other studies (420 children investigated in Menorca Island, Spain, and 3207 children studied in Great Britain) find no association between prenatal smoking exposure in mothers and DCD in children^18,19^. The inconsistent results presented above emphasize the urgent need to explore the association between prenatal smoking in mothers and DCD in children with a larger study population size.

China is the largest producer and consumer of tobacco in the world, with 300 million adult smokers, and 1 million deaths attributed to tobacco consumption each year^20–22^. In China, the prevalence of active smoking (firsthand smoke, FHS) among pregnant women ranges from 0.7% to 1.6%, but 38.9% to 75.1% of non-smoking women are exposed to secondhand smoke (SHS)^23^. Moreover, one observational study evaluating the urinary cotinine concentration in Chinese pregnant women indicates that the prevalence of urinary cotinine detection reaches 87%, indicating that pregnant women are at high risk of being exposed to SHS^24^, but evidence on the relationship between DCD in children and SHS as well as FHS in pregnant women is still limited in China.

We conducted a cross-sectional study in Shanghai, China with the aim to understand the prevalence of prenatal SHS and FHS exposure among mothers, as well as the prevalence of DCD among children aged 3–6 years, and to explore if mothers’ prenatal tobacco smoke exposure is positively associated with the occurrence of DCD among children aged 3–6 years.

## METHODS

### Study population

The cross-sectional study was conducted during March and September in 2018 in the Songjiang district of Shanghai. We employed a cluster sampling method to recruit the study population. First, we randomly selected 20 out of the 115 kindergartens in Songjiang District. Then, we recruited all children aged 3–6 years and their mothers from each of the 20 selected kindergartens, and a questionnaire interview was completed on-site in the selected kindergartens. Overall, a total of 8626 children and their mothers were sampled and invited to participate in the study. The ethics approval was obtained from Songjiang Maternal and Childcare Hospital Institution Review Board (IRB#20171203). An informed consent form, both for the children and their mothers, was orally communicated face-to-face by study coordinators and then signed by each mother before the questionnaire interview, and information that could identify individual participants during or after data collection could not be accessed by authors. Finally, 8586 children and their mothers completed the interview and were included in the analysis.

### Data collection

The questionnaire for data collection in this study was designed by Songjiang Maternal and Children’s Health-care Hospital with reference to a published questionnaire in Shanghai, China^6^. A pilot study demonstrated that the split-half reliability coefficient of the questionnaire was 0.81 and the content validity coefficient was 0.84. The questionnaire includes four parts. Part A covered 10 questions on demographic information (gender of children, age of children, age of mother, education of mother, family yearly income etc.). Part B covered 9 items on prenatal tobacco smoke exposure information and perinatal factors of DCD : ‘Did you smoke cigarettes during your pregnancy?’, ‘Did anyone smoke cigarettes around you at home or in the workplace during your pregnancy?’, ‘What was the delivery method of your child, vaginal or cesarean delivery?’, ‘Was your child delivered before the gestational week of 37?’, ‘Did fetal asphyxia happen to your child during the delivery?’, ‘Was your child admitted to the ICU after the delivery?’, ‘How long did you breastfeed your child?’, etc.). Part C covered 15 items on daily physical activity information of the children. Part D covered personal contact information both for the investigator and the mother. Data from this study are available upon request from the corresponding author. The request should state the title and aim of the research for which the data are being requested.

### Definition and index calculation

We defined prenatal exposure to firsthand smoke (FHS) for a woman who smoked at least one cigarette every day for at least six months during the pregnancy^20,21^, while prenatal exposure to secondhand smoke (SHS) is defined for a non-smoker woman exposed to tobacco smoke for at least 10 minutes per day in the workplace or at home during the pregnancy^23^. The prevalence of prenatal exposure to FHS was calculated as the number of women who prenatally were exposed to FHS divided by the total number of participants, and the prevalence of prenatal exposure to SHS was calculated as the number of women who prenatally were exposed to SHS divided by the total number of participants. We designed two steps to diagnose DCD among children in this study. In Step 1, we applied the movement assessment battery for children-second edition (MABC-2) to evaluate the developmental coordination abilities by scoring^25,26^. In Step 2, we invited an experienced pediatrician to confirm the DCD diagnosis among children with a score of 71 or lower that were evaluated in Step 1. The prevalence of DCD was calculated as the number of children with DCD divided by the total number of children. In this study, we defined preterm birth as a baby born alive before 37 gestational weeks. The age of mother was classified into four age groups (19–25, 26–30, 31–35, and 36–48 years). Education of mother was recorded as completed years of schooling and categorized into four categories: 0–6 years (illiterate or primary school), 7–9 years (junior high school), 10–12 years (senior high school), and >12 years (college and above). Family yearly income (in equivalent US$) was classified into five groups: <8000, 8000–16000, 16001–24000, 24001–48000 and >48000 US$ equivalent.

### Data analysis

Data analysis was performed by SAS software (version 9.2). We described the data by using frequency counts and proportions (prevalence) for qualitative variables and means with standard deviations (SD) for quantitative variables. We applied the chi-squared test to examine the prevalence difference of prenatal exposure to FHS as well as SHS among mothers with different demographic characteristics. We applied logistic regression to calculate the odds ratios (ORs) and 95% confidence interval (95% CI) of DCD prevalence among children with different age and gender, as well as among the mothers with different demographic characteristics. A univariate logistic regression and multivariate logistic regression with adjustment for potential confounders were applied to explore the association of mothers’ prenatal tobacco smoke exposure, perinatal factors and the DCD prevalence among the children aged 3–6 years. Potential confounders were identified by using directed acyclic graphs (DAGs) method. Figures were produced to explore whether the prenatal tobacco smoke exposure among mothers was positively associated with a higher prevalence of DCD among their children by gender and by age. In this study, a p<0.05 (two-tailed) was considered statistically significant.

## RESULTS

In this study, 8586 children qualified, of which 4595 were boys (53.52%), with overall age range from 3 to 6 years and average age 4.46 ± 0.96 years. The age of mothers ranged from 19 to 48 years with average age 32.17 ± 3.95 years. The majority of mothers (71.13%) had college education and above (>12 years), and nearly half of the investigated families had yearly income over 48000 US$, while 52.94% of the mothers were local residents.

### Prevalence of prenatal tobacco smoke exposure

Among 8586 mothers, the prevalence of prenatal FHS exposure (active smoking) was 0.94% (81/8586). The prevalence of prenatal FHS exposure was significantly higher in mothers of local residents (1.23%) than no-local residents (0.62%). Mothers aged <25 years (2.53%) or >40 years (1.30%) had a higher prevalence of prenatal FHS exposure. Mothers with college education and above had a lower prevalence of prenatal FHS exposure than mothers who were illiterate, had primary or high school education (junior and senior) (Table 1).

**Table 1:** The prevalence of prenatal exposure to firsthand and secondhand tobacco smoke among mothers whose children were aged 3–6 years, rural area of Shanghai, China, 2018 (N=8586)

*Variables*	*Participants (N=8586)*		*Firsthand smoke (N=81)*		*Secondhand smoke (N=1785)*	

	*n*	*(%)^a^*	*n*	*(%)^b^*	*n*	*(%)^b^*
**Age** (years)^c,d^						
19–25	237	2.76	6	2.53	62	26.16
26–30	2870	33.43	25	0.87	728	25.37
31–35	3858	44.93	29	0.75	730	18.92
36–48	1621	18.88	21	1.30	265	16.35
**Education^c,d^**						
Illiterate/Primary	71	0.83	1	1.41	14	19.72
Junior High	795	9.26	14	1.76	181	22.77
Senior High	1613	18.79	25	1.55	373	23.12
College and above	6107	71.13	41	0.67	1217	19.93
**Family yearly income** (US$)^d^						
<8000	1344	15.65	15	1.12	334	24.85
8000–16000	1206	14.05	9	0.75	273	22.64
16001–24000	1117	13.01	12	1.07	239	21.40
24001–48000	1169	13.62	11	0.94	204	17.45
>48000	3750	43.68	34	0.91	735	19.60
**Resident status^c,d^**						
Local	4545	52.94	56	1.23	1094	24.07
Non-local	4041	47.06	25	0.62	691	17.10

a Proportion.

b Prevalence.

c The differences between group on prevalence of prenatal exposure to firsthand sm ke were statistically significant (p<0.01).

d The differences between group on prevalence of prenatal exposure to secondhand smoke were statistically significant (p<0.01).

In all, 1785 out of 8586 mothers were exposed to SHS during pregnancy with a prevalence of prenatal SHS exposure of 20.79%. The prevalence of prenatal SHS exposure was significantly higher in mothers aged <30 years (25.43%) than mothers aged >30 years (18.16%). Mothers who were local residents had higher prevalence of prenatal SHS exposure (24.07%) than mothers who were no-local residents (17.10%). Mothers with high school education (junior and senior) had higher prevalence of prenatal SHS exposure than other mothers, and mothers with a higher family yearly income had a lower prevalence of prenatal SHS exposure (Table 1).

### Prevalence of developmental coordination disorder (DCD) in children

In this study, 571 children were finally confirmed as having developmental coordination disorder (DCD), the prevalence of DCD was 6.65%. The prevalence of DCD was significantly higher in boys (7.64%) than in girls (5.51%) (OR=1.42; 95% CI: 1.19–1.69). Children aged 3 years had higher prevalence of DCD (9.77%) than children aged 4, 5 or 6 years (7.44%, 5.27% and 4.28%, respectively) (Table 2).

**Table 2:** The prevalence of developmental coordination disorder (DCD) among children aged 3–6 years, rural area of Shanghai, China, 2018 (N=8586)

*Variables*	*n (%)*	*DCD*		*OR*	*95% CI*	*AOR*	*95% CI*

		*Yes*	*No*				
**Gender**							
Female	3991 (46.68)	220 (5.51)	3771 (94.49)	1.00	-	-	-
Males	4595 (53.52)	351 (7.64)	4244 (92.36)	1.42	1.19–1.69	-	-
**Age** (years)^a^							
3	1525 (17.76)	149 (9.77)	1376 (90.23)	1.00	-	1.00	-
4	2930 (34.13)	218 (7.44)	2712 (92.56)	0.74	0.59–0.92	0.72	0.58–0.90
5	2752 (32.05)	145 (5.27)	2607 (94.73)	0.52	0.41–0.65	0.49	0.38–0.62
6	1379 (16.06)	59 (4.28)	1320 (95.72)	0.41	0.30–0.56	0.37	0.27–0.51
**Age of mother**^b^ (years)							
19–25	237 (2.76)	31 (13.08)	206 (86.92)	1.00	-	1.00	-
26–30	2870 (33.43)	205 (7.14)	2665 (92.86)	0.51	0.34–0.77	0.74	0.48–1.11
31–35	3858 (44.93)	223 (5.78)	3635 (94.22)	0.41	0.27–0.61	0.74	0.48–1.13
36–48	1621 (18.88)	112 (6.91)	1509 (93.09)	0.49	0.32–0.75	0.78	0.50–1.21
**Education of mother^b^**							
Illiterate/Primary	71 (0.83)	12 (16.90)	59 (83.10)	1.00	-	1.00	-
Junior High	795 (9.26)	112 (14.09)	683 (85.91)	0.81	0.42–1.55	0.85	0.44–1.65
Senior High	1613 (18.79)	148 (9.18)	1465 (90.82)	0.50	0.26–0.95	0.52	0.27–1.01
College and above	6107 (71.13)	299 (4.90)	5808 (95.10)	0.25	0.14–0.48	0.27	0.14–0.52
**Family yearly income** (US$)^c^							
<8000	1344 (15.65)	141 (10.49)	1203 (89.51)	1.00	-	1.00	
8000–16000	1206 (14.05)	77 (6.38)	1129 (93.62)	0.58	0.44–0.78	0.69	0.52–0.93
16001–24000	1117 (13.01)	59 (5.28)	1058 (94.72)	0.48	0.35–0.65	0.62	0.45–0.85
24001–48000	1169 (13.62)	51 (4.36)	1118 (95.64)	0.39	0.28–0.54	0.54	0.38–0.75
>48000	3750 (43.68)	243 (6.48)	3507 (93.52)	0.59	0.48–0.74	0.69	0.55–0.86
**Resident status^d^**							
Non-local	4041 (47.06)	278 (6.88)	3763 (93.12)	1.00	-	-	
Local	4545 (52.94)	293 (6.45)	4252 (93.55)	0.93	0.79–1.11	0.86	0.72–1.03

a AOR adjusted for the age of mother and education of mother.

b AOR adjusted for the age of children, education of mother, family income and residency status.

c AOR adjusted for the age of mother, education of mother and residency status.

d AOR adjusted for the age of mother, education of mother and family income. AOR: adjusted odds ratio.

In comparison with children whose mothers were aged <25 years, the prevalence of DCD was lower than among those whose mothers were aged 26–30 years (OR=0.51; 95% CI: 0.34–0.77), 31–35 years (OR=0.41; 95% CI: 0.27–0.61) or 36–48 years (OR=0.49; 95% CI: 0.32–0.75). Similarly, the prevalence of DCD was significantly lower among children whose mothers had an education of senior high school (OR=0.50; 95% CI: 0.26–0.95) or college and above (OR=0.25; 95% CI: 0.14–0.48) compared with those children whose mothers were lower educated. With increase in family income, the prevalence of DCD in children decreased significantly (Table 2).

### Relationship between DCD in children and prenatal tobacco smoke exposure in mothers

Mothers’ prenatal tobacco smoke exposure was positively associated with the occurrence of DCD in children. Children whose mothers were exposed to FHS prenatally had significantly higher odds of DCD prevalence than children whose mothers were without prenatal FHS exposure (OR=4.42; 95% CI: 2.62–7.44), even after adjustment of prenatal or perinatal confounders (OR=3.68; 95% CI: 2.17–6.25). Likewise, children whose mothers were prenatally exposed to SHS had higher odds of DCD prevalence than children whose mothers were without prenatal SHS exposure (OR=1.77; 95% CI: 1.47–2.14), even after adjustment of prenatal or perinatal confounders (OR=1.63; 95% CI: 1.35–1.97) (Table 3).

**Table 3:** The correlation of mothers’ prenatal tobacco smoke exposure, perinatal factors and the prevalence of DCD among their children aged 3–6 years, rural area of Shanghai, China 2018 (N=8586)

*Variables*	*DCD*		*Univariate*		Multivariate*	

	*Yes n (%)*	*No n (%)*	*OR*	*OR 95% CI*	*OR*	*OR 95% CI*
**Prenatal exposure to firsthand smoke**						
Yes	19 (23.46)	62 (76.54)	4.42	2.62–7.44	3.68^a^	2.17–6.25
No	552 (6.49)	7953 (93.51)	1.00	–	1.00	–
**Prenatal exposure to secondhand smoke**						
Yes	176 (9.86)	1609 (90.14)	1.77	1.47–2.14	1.63^b^	1.35–1.97
No	395 (5.81)	6406 (94.19)	1.00	–	1.00	–
**Children with preterm birth**						
Yes	78 (8.78)	810 (91.22)	1.41	1.10–1.81	1.28^c^	0.99–1.67
No	493 (6.40)	7205 (93.60)	1.00	–	1.00	–
**Children’s delivery method**						
Vaginal	236 (6.28)	3519 (93.72)	1.11	0.94–1.32	1.09^d^	0.92–1.30
Cesarean	335 (6.93)	4496 (93.07)	1.00	–	1.00	–
**Fetal asphyxia in delivery**						
Yes	34 (15.11)	191 (84.89)	2.59	1.78–3.77	2.34^e^	1.60–3.43
No	537 (6.42)	7824 (93.58)	1.00	–	1.00	–
**Newborn infants treated in ICU**						
Yes	70 (8.56)	748 (91.44)	1.36	1.01–1.76	1.12^f^	0.85–1.47
No	501 (6.46)	7267 (93.55)	1.00	–	1.00	–
**Children’s breastfeeding time** (months)						
<6	204 (7.96)	2360 (92.04)	1.33	1.12–1.59	1.30^g^	1.08–1.56
≥6	367 (6.09)	5655 (93.91)	1.00	–	1.00	–

* Multivariate logistic regression with covariates adjusted during the logistic regression selected by Directed Acyclic Graph (DAG) method.

a OR after covariate adjustment of prenatal exposure of secondhand smoke and breastfeeding time.

b OR after covariate adjustment of prenatal exposure of firsthand smoke, fetal asphyxia in delivery, newborn infants treated in ICU, family yearly income and breastfeeding time.

c OR after covariate adjustment of fetal asphyxia in delivery, newborn infants treated in ICU, breastfeeding time, age of children, gender of children and family yearly income.

d OR after covariate adjustment of prenatal exposure of secondhand smoke, preterm birth, breastfeeding time, age and gender of children.

e OR after covariate adjustment of prenatal exposure of secondhand smoke, preterm birth, newborn infants treated in ICU, and gender of children.

f OR after covariate adjustment of prenatal exposure of secondhand smoke, preterm birth, fetal asphyxia in delivery, breastfeeding time, a d age of children.

g OR after covariate adjustment of prenatal exposure of secondhand and firsthand smoke, preterm birth, newborn infants treated in ICU, and age of children.

In this study, children with fetal asphyxia during delivery had higher odds of DCD prevalence (OR=2.34; 95% CI: 1.60–3.43), and children breastfeeding for less than 6 months also had higher odds of DCD prevalence (OR=1.30; 95% CI: 1.08–1.56).

**Figure 1:**
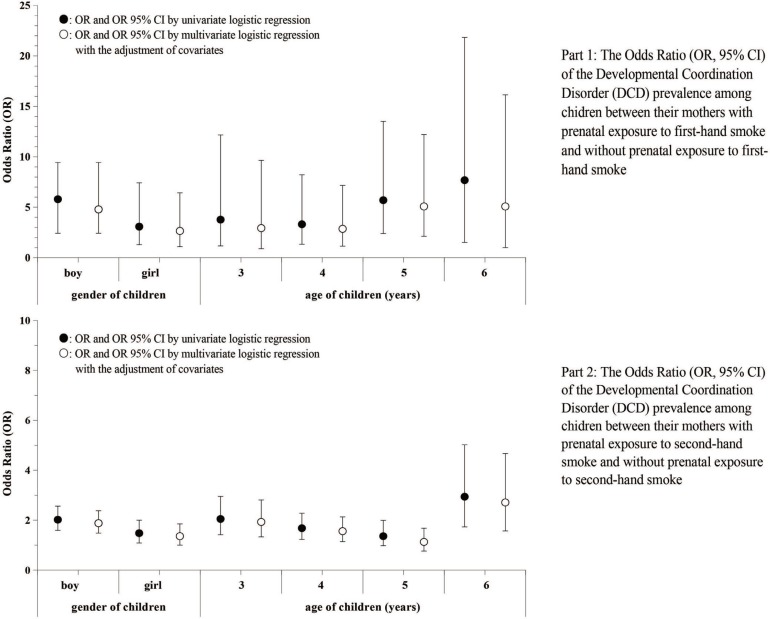
The Odds Ratio (OR, 95% CI) of the Developmental Coordination Disorder (DCD) prevalence among children between mothers with prenatal exposure to firsthand smoke and without prenatal exposure to firsthand smoke, as well as between mothers with prenatal exposure to secondhand smoke and without prenatal exposure to secondhand smoke

Figure 1 indicates that the association between mothers’ prenatal tobacco smoke exposure and DCD in children was stronger in boys than in girls, both for FHS exposure (boys: OR=5.8; girls: OR=3.08) and SHS exposure (boys: OR=2.02; girls: OR=1.48). Stronger associations were also identified among children aged 6 years than children aged 3–5 years, both for the FHS exposure (OR=7.68; 95% CI: 1.52–21.83) and SHS exposure (OR=2.94; 95% CI: 1.73–5.02) (Figure 1).

## DISCUSSION

Tobacco smoke contains many harmful substances and is one of the most ubiquitous environmental health hazards both for adults and children^27^. Prenatal exposure to tobacco smoke is reported to be associated with higher risk of still birth, congenital malformation, lower birthweight, and decreased head circumference at birth^28,29^. In this study, the prevalence of prenatal FHS exposure (active smoking) was 0.94%, which was lower than the findings of the GATS^23^ in 2012 and SSACB in 2017 cohorts^20–22^. Meanwhile, the prevalence of prenatal SHS exposure among women was 20.79%, which was lower than the investigation results in Sichuan Province (70%) and Henan Province (60%), as well as in the GATS investigation in China (53%) and Indonesia (76%)^23,30,31^. The lower prevalence of prenatal tobacco smoke exposure might be attributable to the implementation of public health education and intervention on smoking cessation in Shanghai^32^. Meanwhile, many more residents are aware of the physical harm of tobacco use in recent years and have stopped tobacco smoking successfully^33^. Whereas we still notice a high prevalence of prenatal SHS exposure among mothers <30 years with education of junior/senior high school, thus we suggest tobacco control measures should be focused on their living and working circumstances in the future.

Currently, DCD is identified as an impairment of motor ability, which is not just relevant to the movement system but related to a complex series of neurological dysfunctions involving memory, attention, executive function and reasoning^34^. In this study, the prevalence of DCD among children was 6.65%, which is consistent with previous studies^5–7^, but slightly lower than the findings in Suzhou (8.3%) in 2012. The lower DCD prevalence might be attributed to the combined effect of MABC-2 application and pediatrician diagnosis, which decreased the false positive rate of DCD screening by the tools of DCDQ and DSM-5 in the Suzhou study^26^. Moreover, the prevalence of DCD in this study was higher among boys than girls, and the prevalence of DCD decreased when children became older, these findings are in line with previous investigations^10,11^. We suggest that DCD screening should be implemented among preschool children aged 3–6 years and primary school children aged 7–12 years, especially among boys, which could provide an opportunity for DCD early intervention.

Previous studies demonstrated that the risk of DCD increases among children from less advantaged socioeconomic backgrounds, among children with a birthweight under 2500 g or preterm birth (<37 weeks of gestation)^2,8,9^. In this study, we identified a higher prevalence of DCD among children with preterm birth, fetal asphyxia in delivery, a history of perinatal ICU admission and breastfeeding less than 6 months, whereas significant associations were only observed between higher DCD prevalence and children with fetal asphyxia in delivery or with breastfeeding time less than 6 months after the adjustment for potential confounding factors, consistent with previous studies^2,9,10^. But the possible causal association and pathogenic mechanism between the initiation of DCD and the fetal asphyxia in delivery as well as the short breastfeeding time is still unclear and needs to be clarified in the future.

In this study, we observed an elevated prevalence of DCD in children whose mother was exposed to prenatal tobacco smoke, both for FHS and SHS. The prevalence of DCD was 4.42 times higher among children if their mothers were exposed to FHS prenatally, and 1.77 times higher among children if their mothers were exposed to SHS prenatally. Previous studies indicated that the association between high prevalence of DCD in children and mothers’ prenatal tobacco exposure was confounded by social, economic factors, as well as perinatal risk factors including preterm birth, lower birthweight, gender of children, age of children, etc^8,10,19^. In order to address this, we adjusted the potential confounding factors mentioned above, ascertained by directed acyclic graphs (DAGs)^16,20^, and logistic regression analysis with covariable adjustment demonstrated that children still had higher risk of DCD occurrence if their mother was exposed to tobacco smoke prenatally, with OR=3.68 and OR=1.63 for FHS and SHS exposure, respectively. Potential mechanisms underlying this association include intrauterine hypoxia, and direct toxic effects of nicotine^35^. Nicotine is the major teratogenic component of tobacco smoke and could lead to adverse neurodevelopmental consequences. Other potential mechanisms include a form of fetal malnutrition due to maternal tobacco smoke exposure and the possible influence of components in cigarette smoke other than nicotine^19,35^, but mechanisms of the association need to be clarified in further studies.

### Strengths and limitations

This study is the first attempt to estimate the association of prenatal FHS and SHS exposure among mothers and occurrence of DCD in children aged 3–6 years in China. A key strength of this study is the large sampling population size. We sampled 8586 children accounting for about 20% of the total population of preschool children in Songjiang district, and hence the study results could be generalized to all children aged 3–6 years in Songjiang district or even to other rural areas of Shanghai. Another strength of this study is that we used directed acyclic graphs (DAGs) to identify potential confounding factors and adjusted them in the multivariate logistic regression analysis, which ensured a relatively unbiased association between children’s DCD prevalence and mothers’ prenatal tobacco smoke exposure.

Our study has some limitations. First, the cross-sectional study design only allows the calculation of prevalence and may induce some information bias, and the positive association between prenatal tobacco exposure in mothers and DCD in children should be validated by studies with a design of case-control or cohort. Second, detailed information of prenatal SHS and FHS exposure (daily tobacco smoking, hours of SHS exposure at home and workplace etc.) among mothers was not recorded, making it impossible to estimate the dose-effect relationship between DCD in children and prenatal tobacco smoke exposure in mothers. Third, information of perinatal determinants of DCD is self-reported, which might lead to some recall bias, and the prenatal SHS and FHS exposure among mothers might be under-reported, leading to a potential risk of under-estimating the odds ratios. The incorporation of some improvements should be considered in further follow-up studies.

## CONCLUSIONS

The occurrence of developmental coordination disorder (DCD) among children is positively associated with prenatal tobacco smoke exposure among their mothers, both for FHS and SHS, but the causality needs to be validated by future prospective studies. We emphasize that it is crucial to implement tobacco control measures to decrease the prevalence of smoking among pregnant women and also among their family members and fellows at work in Shanghai, China.

## References

[1] Cheng YTY, Wong TRN, Tsang WWN (2019). Neuromuscular training for children with developmental coordination disorder: A randomized controlled trial. Medicine (Baltimore).

[2] Rikke FL, Laust HM, Torben M, Anne-Marie NA (2013). Determinants of developmental coordination disorder in 7 year old children: a study of children in the Danish National Birth Cohort. Dev Med Child Neurol.

[3] Fong SSM, Lee VYL, Pang MYC (2011). Sensory organization of balance control in children with developmental coordination disorder. Res Dev Disabil.

[4] Fong SSM, Lee VYL, Chan NNC, Chan RS, Chak WK, Pang MY (2011). Motor ability and weight status are determinants of out-of-school activity participation for children with developmental coordination disorder. Res Dev Disabil.

[5] Lingam R, Hunt L, Golding J, Jongmans M, Emond A (2009). Prevalence of developmental coordination disorder using the DSM-IV at 7 years of age: a UK population-based study. Pediatrics.

[6] Hua J, Gu GX, Zhu QQ (2015). The reliability and validity of the Developmental Coordination Disorder questionnaire’ 07 for children aged 4-6 years in mainland China. Res Dev Disabil.

[7] Arpino C, Compagnone E, Montanaro ML (2010). Preterm birth and neurodevelopmental outcome: a review. Childs Nerv Syst.

[8] Roberts G, Anderson PJ, Davis N, De Luca C, Cheong J, Doyle LW (2011). Developmental coordination disorder in geographic cohorts of 8-year-old children born extremely preterm or extremely low birthweight in the 1990s. Dev Med Child Neurol.

[9] Davis NM, Ford GW, Anderson PJ, Doyle LW (2007). Developmental coordination disorder at 8 years of age in a regional cohort of extremely-low-birthweight or very preterm infants. Dev Med Child Neurol.

[10] Goyen TA, Lui K (2009). Developmental coordination disorder in ‘apparently normal’ school children born extremely preterm. Arch Dis Child.

[11] Rodriguez A, Bohlin G (2005). Are maternal smoking and stress during pregnancy related to ADHD symptoms in children?. J Child Psychol Psychiatry.

[12] Batstra L, Hadders-Algra M, Neeleman J (2003). Effect of antenatal exposure to maternal smoking on behavioural problems and academic achievement in childhood: prospective evidence from a Dutch birth cohort. Early Human Dev.

[13] McLeod KR, Langevin LM, Goodyear BG, Dewey D (2014). Functional connectivity of neural motor networks is disrupted in children with developmental coordination disorder and attention deficit/hyperactivity disorder. Neuroimage Clin.

[14] Wulaningsih W, Serrano FE, Utarini A (2016). Smoking, second-hand smoke exposure and smoking cessation in relation to leukocyte telomere length and mortality. Oncotarget.

[15] Liu H, Chen Q, Lei L (2018). Prenatal exposure to perfluoroalkyl and polyfluoroalkyl substances affects leukocyte telomere length in female newborns. Environ Pollut.

[16] Mahlberg N, James ME, Bulten R, Rodriguez C, Kwan M, Cairney J (2019). Investigating the association between exposure to second hand smoke in utero and developmental coordination disorder. Front Pediatr.

[17] Slykerman RF, Thompson JM, Clark PM (2007). Determinants of developmental delay in infants aged 12 months. Paediatr Perinat Epidemiol.

[18] Julvez J, Ribas-Fito N, Torrent M, Forns M, Garcia-Esteban R, Sunyer J (2007). Maternal smoking habits and cognitive development of children at age 4 years in a population-based birth cohort. Int J Epidemiol.

[19] Larsson M, Montgomery SM (2011). Maternal smoking during pregnancy and physical control and coordination among offspring. J Epidemiol Community Health.

[20] Wang RP, Jiang YG, Yao CX (2019). prevalence of tobacco related chronic diseases and its role in smoking cessation among smokers in a rural area of Shanghai, China: a cross sectional study. BMC Public Health.

[21] Wang RP, Li B, Jiang YG, Guan Y, Wang G, Zhao G (2019). Smoking cessation mutually facilitates alcohol drinking cessation among tobacco and alcohol co-users: A cross-sectional study in a rural area of Shanghai, China. Tob Induc Dis.

[22] Wang RP, Jiang YG, Li X (2019). Relationships between smoking duration, smoking intensity, hypothetical tobacco price increases, and smoking habit change intention among current smokers in Shanghai. J Int Med Res.

[23] Liu BQ, Song LL, Zhang L (2019). Prenatal second hand smoke exposure and newborn telomere length. Pediatr Res.

[24] Xiao X, Li Y, Song XX (2018). Discrepancy between self reported and urine cotinine verified environmental tobacco smoke exposure among rural pregnant women in China. Int J Environ Res Public Health.

[25] Hua J, Jin H, Gu G, Liu M, Zhang L, Wu Z (2014). The influence of Chinese one-child family status on Developmental Coordination Disorder status. Res Dev Disabil.

[26] Lee KJ, Jung TY, Lee DK (2019). A comparison of using the DSM-5 and MABC-2 for estimating the developmental coordination disorder prevalence in Korean children. Res Dev Disabil.

[27] Line HC, Birgit BH, Henning SP (2019). Prenatal smoking exposure, measured as maternal serum cotinine, and children’s motor developmental milestones and motor function: A follow-up study. Neurotoxicology.

[28] Leonardi-Bee J, Britton J, Venn A (2011). Secondhand smoke and adverse fetal outcomes in nonsmoking pregnant women: a meta-analysis. Pediatrics.

[29] Windham GC, Abigail E, Barbara H (1999). Evidence for an association between environmental tobacco smoke exposure and birthweight: a meta-analysis and new data. Pediatric and Perinatal Epidemiology.

[30] Soesanti F, Uiterwaal CSPM, Grobbee DE, Hendarto A, Dalmeijer GW, Idris NS (2019). Antenatal exposure to second hand smoke of non-smoking mothers and growth rate of their infants. PloS One.

[31] Zhang L, Hsia J, Tu X (2015). Exposure to second-hand tobacco smoke and interventions among pregnant women in China: A systematic review. Prev Chronic Dis.

[32] Liu Y, Song HJ, Wang TY (2018). Determinants of tobacco smoking among rural to urban migrant workers: a cross-sectional survey in Shanghai. BMC Public Health.

[33] Pek KI, Iona YM, Guo Y (2019). Patterns and trends of alcohol consumption in rural and urban areas of China: findings from the China Kadoorie Bio-bank. BMC Public Health.

[34] Ke L, Duan W, Xue Y, Wang Y (2019). Developmental coordination disorder in Chinese children is correlated with cognitive deficits. Front Psychiatry.

[35] Kiechl-Kohlendorfer U, Ralser E, Peglow UP, Reiter G, Griesmaier E, Trawöger R (2010). Smoking in pregnancy: a risk factor for adverse neurodevelopmental outcome in preterm infants?. Acta Padiatr.

